# An Adult Kawasaki Disease with Coronary Artery Involvement: A Unique Case Report and Literature Review

**DOI:** 10.7759/cureus.5529

**Published:** 2019-08-30

**Authors:** Mohammed FaisalUddin, Pulwasha M Iftikhar, Azadeh Khayyat, Javidulla Khan, Azeem H Arastu

**Affiliations:** 1 Internal Medicine, Deccan College of Medical Sciences, Hyderabad, IND; 2 Obstetrics and Gynecology, St. John's University, New York, USA; 3 Internal Medicine, Ahvaz Jundishapur University of Medical Sciences, Ahvaz, IRN

**Keywords:** kawasaki disease, coronary artery aneursym, vasculitis, artharlgia, rash, gamma globulin

## Abstract

Kawasaki disease (KD) is an acute, febrile, vasculitis of mainly large to medium-sized vessels. KD is a self-limited illness of infancy and childhood and 80% of the patients are younger than four years of age with an incidence of 5.6/100,000 in the United States. We present an unusual case of an 18-year-old man with several unique features of KD. He was admitted to the hospital with fever, rash, and arthralgia for one week. KD was among the differentials for fever, rash, and arthralgia. Later all the laboratory diagnosis for bacterial and viral infections including blood and urine culture came out negative and he was further evaluated for KD with electrocardiography (EKG), echocardiography, and angiography which showed myocarditis. Based on typical features of fever, rash, arthralgia, bilateral conjunctival injection, cervical lymphadenopathy, and prominent tongue papillae he was diagnosed with KD.

## Introduction

Kawasaki disease (KD) is an acute vasculitis of large to medium-sized vessels. KD is a self-limited illness of infancy and childhood and 80% of the patients are younger than four years of age with an incidence of 5.6/100,000 in the United States [1]. It is rarely found in adults and its clinical significance stems from the involvement of coronary arteries leading to aneurysm formation that can rupture or thrombose, resulting in myocardial infarction [2-4]. In genetically vulnerable persons, a variety of infectious agents (mostly viral) have been posited to trigger the disease. The acute vasculitis typically subsides spontaneously or in response to treatment, but aneurysm formation due to wall damage can remain and cause devastating outcomes. As with other vasculitis syndromes, healed lesions also can exhibit obstructive intimal thickening. Pathologic changes outside the cardiovascular system are rare and not significant.

KD typically manifests with erythema of the palms and soles, conjunctival and oral erythema, edema of the hands and feet, a desquamative rash, and cervical lymph node enlargement. Approximately 20% of untreated patients develop cardiovascular sequelae, ranging from asymptomatic coronary arteritis to coronary artery ectasia and large coronary artery aneurysm which eventually rupture or get thrombosed leading to infarction, and sudden death. The diagnostic criteria for KD as defined by the Centers for Disease Control and Prevention (CDC) include an unexplained fever lasting five days or more and at least four out of the five following criteria. 1) polymorphous exanthema; 2) changes in the peripheral extremities, erythema or indurative edema of the palms and soles (acute phase) or desquamation around the fingertips (convalescent phase); 3)bilateral nonexudative conjunctival injection; 4) changes in the oropharynx, that is, injected or fissured lips, strawberry tongue, and injected pharynx; and 5) acute nonsuppurative cervical lymphadenopathy. Patients having less than four of these clinical signs can be diagnosed as having atypical KD [5].

KD has two phases: an acute phase lasting for one to two weeks, followed by a chronic or “convalescent” phase. Common laboratory findings include leukocytosis with a predominance of immature and mature granulocytes, normochromic normocytic anemia, elevated acute-phase proteins, and slightly elevated serum transaminase is a characteristic of the acute phase of KD. Thrombocytopenia may occur in clot formation consumption which is evident by a markedly elevated D-dimer level. Urinalysis may show sterile pyuria in up to 80% of patients. Antinuclear antibody and rheumatoid factor are usually absent. Hypoalbuminemia is associated with more severe acute disease [6-7].

## Case presentation

**Figure 1:**
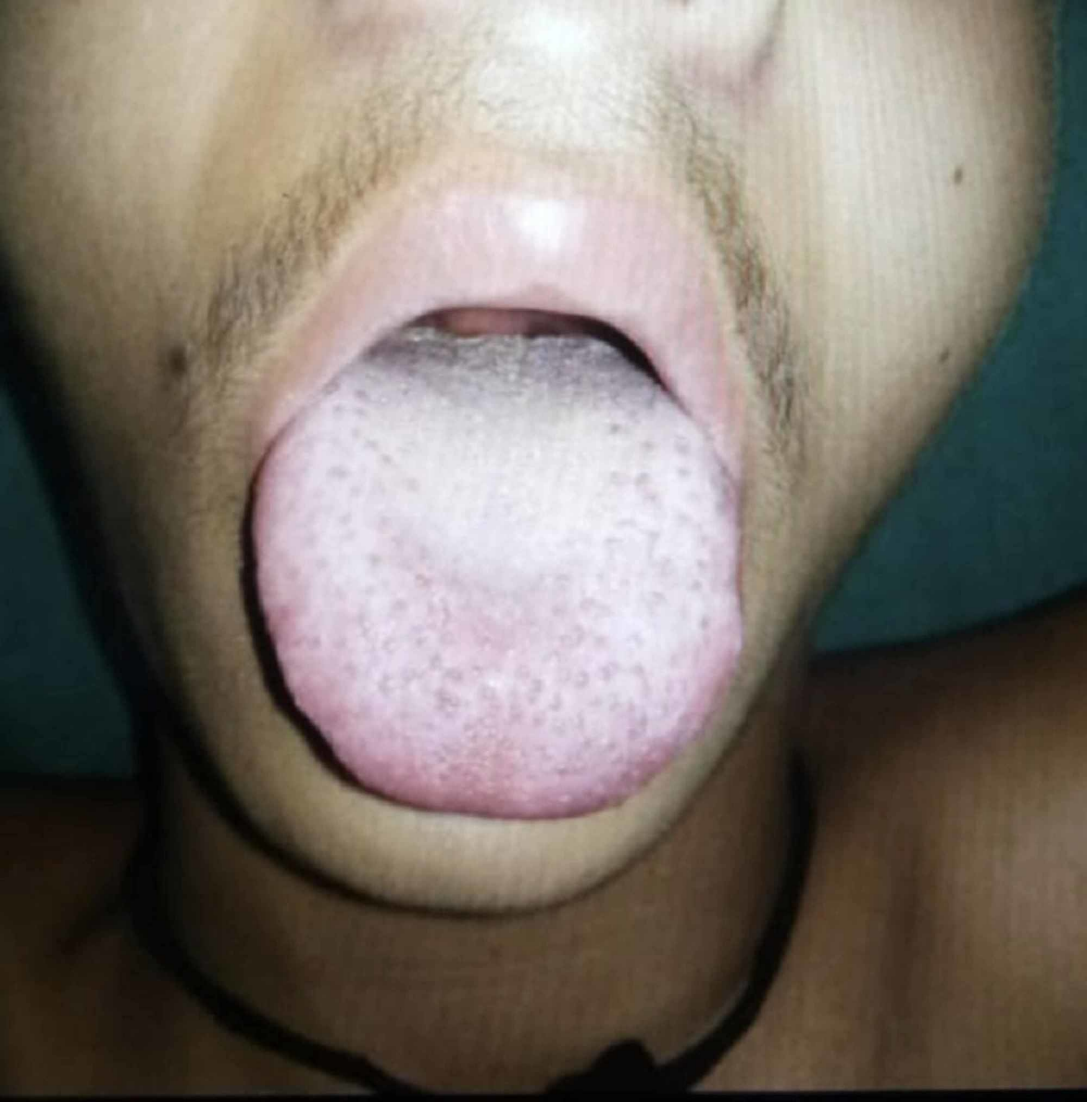
Prominent tongue papillae (strawberry tongue) in adult Kawasaki disease

**Figure 2:**
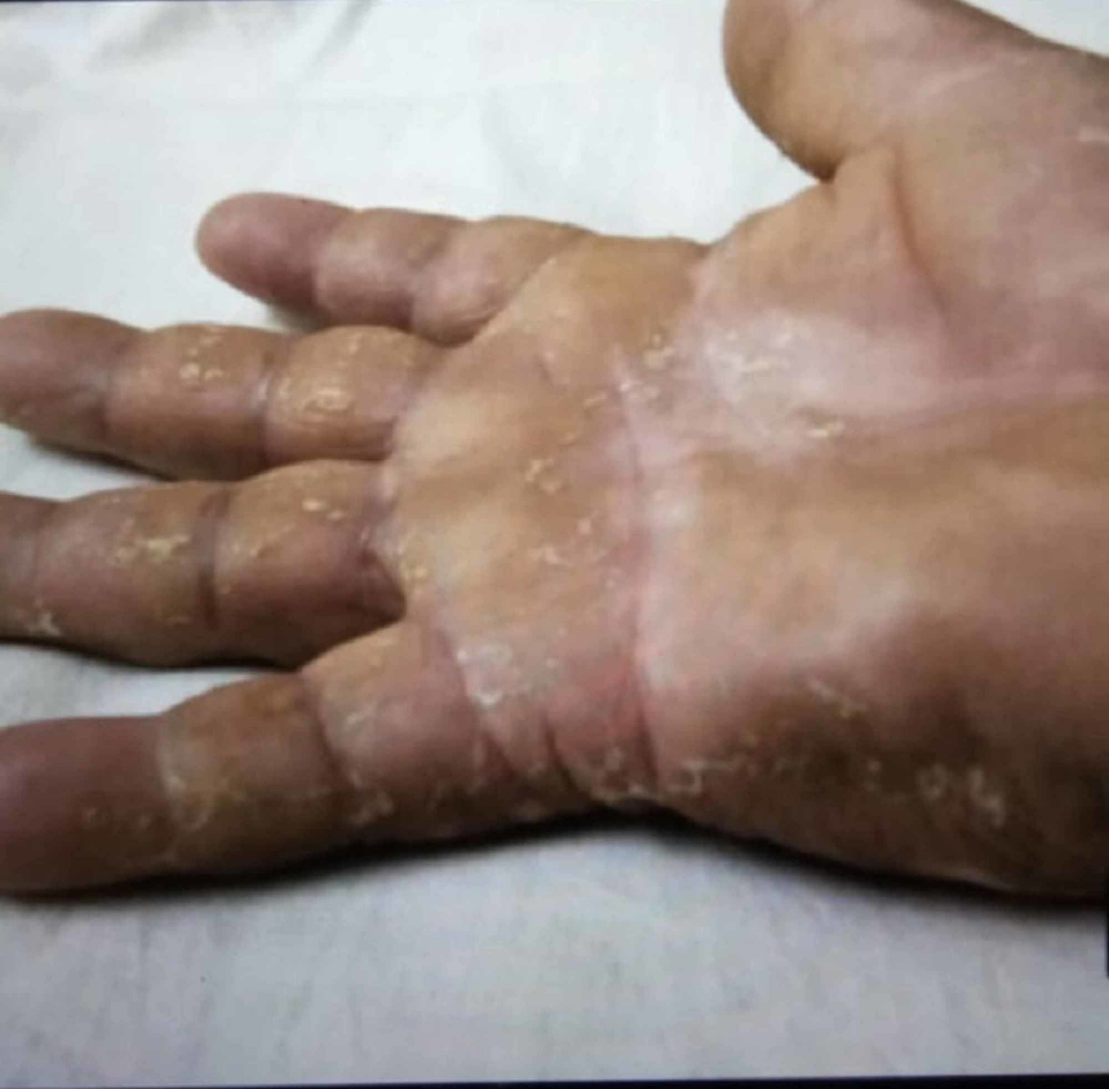
Edematous erythema with desquamation of the palms

An 18-year-old man presented to the emergency department with a one-week history of fever, rash, and arthralgia. He was previously healthy, without any significant medical, surgical and family history. The patient denied any recent chest pain, shortness of breath, orthopnea, dyspnea, bacterial and viral infection. He had no history of intravenous drug use, new sexual partners, tattoos or any animal exposure including contact with domestic dogs, cats, cattle, and deer. On examination, he was febrile (102 F), his blood pressure was 120/80 mm Hg and his pulse was 110/bpm. Head and neck examination revealed injected conjunctiva, dry oral mucositis, fissured lips, and prominent tongue papillae (Figure 1). His physical examination revealed bilateral nonpurulent conjunctivitis and slightly edematous erythema with desquamation of the palms (Figure 2).

On chest auscultation, there was S3 gallop rhythm. The examination of the lungs, abdomen, and neurologic systems were normal. Laboratory test reports revealed anemia with a hemoglobin level of 8.2 g/dl, white blood cell (WBC) count of 14.0×109/l and hypoalbuminemia (2.5 g/dl). He had elevated erythrocyte sedimentation rate of 80 mm/h, C-reactive protein of 98.3 mg/l and alanine aminotransferase of 56 IU/l. Urinalysis showed sterile leukocyturia (30 cells/µl). However, anti-nuclear antibody (ANA), rheumatoid factor, and serum angiotensin-converting enzyme (ACE) levels were normal. His blood and urine cultures and pharyngeal swab culture for Streptococcus pyogenes were negative. Furthermore, serological assays for human immunodeficiency virus, hepatotropic viruses, Epstein-Barr virus, and cytomegalovirus were negative. Chest radiograph was normal but echocardiography showed left ventricular ejection fraction (LVEF) of 37% and angiography was suggestive of myocarditis without aneurysm and stenosis. Electrocardiography (EKG) revealed sinus tachycardia (140-150 bpm). KD was diagnosed based on clinical examination, absence of infection, failure to improve with antibiotic therapy, and the presence of all the signs and symptoms for diagnostic criteria of childhood KD. The patient improved gradually with the use of high dose immunoglobulin (IVIG) infusion according to total body weight (2 g/kg) and oral aspirin (100 mg/kg) divided into four doses, and the dose of aspirin was reduced subsequently. On the seventh day of hospitalization, congestion of the conjunctiva, desquamation of hands, and erythema had resolved. Follow up after two months showed significant clinical improvement in signs and symptoms of the patient. KD should always be considered in the differential diagnosis when an adult patient presents with unexplained fever and a rash.

## Discussion

The complex genetics behind KD has been evaluated and up to 65% of the genetic risk for KD susceptibility may be due to polymorphisms in calcium signaling pathways, the TGFβ pathway, and human leukocyte antigens as reported by some literature. One of the theories postulated for the cause of KD in genetically susceptible patient is infection with a virus leading to activation of both the innate and adaptive immune systems where neutrophils are among the first responder to invade the arterial wall followed by CD8+ T cells, dendritic cells, monocytes/macrophages and eventual activation of the interleukin (IL)-1 and TNF-alpha pathway leading to vessel wall inflammation with subsequent weakening and destruction of elastic lamina and intima leading to formation of aneurysm [8-9].

The arterial lesion develops early in the acute phase of the disease and develops rarely more than four weeks after the disease onset. Coronary artery sequelae are usually the formation of aneurysms in 20% to 25%, seen in 90% of fatal cases. An aneurysm occurs predominantly in the proximal segments and bifurcations of coronary arteries. Coronary artery lesions are dynamic in the late acute and early convalescent phases. The longer the aneurysms or stenotic lesions persist, the less likely they are to resolve. Lesions in the right and left coronary arteries appear to progress differently. Massive thrombosis of the aneurysm is seen predominantly in the right coronary artery, usually within one year after the disease onset. Progressive localized stenosis at the aneurysm inlet or outlet is seen in the left coronary artery, usually within one year after the initial insult. Coronary artery aneurysms are classified as localized or giant measuring >8 mm in diameter and are diagnosed by echocardiography, most of the cases resolve spontaneously and in 50% of cases. Complete resolution is seen on angiography within one year of onset [7-8,10]. Patients with KD-associated aneurysms generally remain asymptomatic and mostly have normal findings on electrocardiograms and stress tests. Overtime aneurysm persists and becomes occlusive increasing the risk of myocardial infarction and even sudden death. Affected arterial segments can get calcified, thrombosed, and stenosed with time. The risk of acquiring myocardial infarction is either early or late after the acute phase. Therefore all the patients should be followed up for the progress of these aneurysms. The most important predictor of subsequent myocardial infarction and other chronic sequelae is aneurysm size where giant aneurysms have a worse outcome as they do not regress and result in ischemic heart disease [11-12]

A study conducted by Inoue O showed that 1,215 patients with coronary artery aneurysms and KD developed giant coronary artery aneurysms in 5% (n=64) patients. Among these 64 patients, only 5% (n=3) regressed without complications. Furthermore, 23% patients (n=15) had myocardial infarction, and 47% patients (n=30) had stenosis [13].

Myocarditis occurs to some degree during the acute phase of KD which resolved completely overtime and seldom affects the left ventricular systolic function in the absence of any coronary lesion. When it occurs clinical findings include an S3 or S4 gallop on physical examination, nonspecific ST-T changes, and cardiomegaly on a chest radiograph. The aortic and mitral valves are rarely involved [12,14]. Alves NR studied 115 patients with KD, 21.7% patients (n=25) had coronary aneurysm, 13.5% patients (n=15) had ophthalmic complications and 33% patients (n=38) had hearing loss. His study showed that KD can progress to permanent and debilitating complications within a few months [14].

No specific diagnostic tests are available for KD, and the diagnosis is based solely on the presence of characteristic clinical findings and recognition of symptoms based on clinical criteria meticulous history-taking and thorough physical examination. The low diagnostic accuracy and masquerading of adult-onset KD, in contrast to that in pediatric patients, can be attributed to the several differential diagnoses that are possible in adult cases. In most of the patients, a disease usually resolves spontaneously after several weeks. Until recently, the standard treatment for acute-phase KD was a four-day regimen of intravenous (IV) gamma globulin (400 mg/kg daily), supplemented with aspirin (100 mg/kg daily divided in four doses). Now, the standard treatment is a single infusion of IV gamma globulin (2 g/kg) followed by low-dose aspirin therapy. The newer regimen is more effective because it safely accelerates the resolution of systemic inflammation. With intravenous immunoglobulin therapy and aspirin, the rate of symptomatic coronary artery disease is reduced to about 4% [15].

## Conclusions

KD should always be considered in the differential diagnosis when an adult patient presents with unexplained fever and a rash as this disease comes with fatal coronary and heart complications leading to poor prognosis and eventually death. Hence, comprehensive cardiac monitoring with echocardiography and angiography should be carried out. Prompt recognition of clinical features of KD by physicians diligently and early institution of IVIG and aspirin can prevent life-threatening cardiac complications.
